# Overview of Chatbots with special emphasis on artificial intelligence-enabled ChatGPT in medical science

**DOI:** 10.3389/frai.2023.1237704

**Published:** 2023-10-31

**Authors:** Chiranjib Chakraborty, Soumen Pal, Manojit Bhattacharya, Snehasish Dash, Sang-Soo Lee

**Affiliations:** ^1^Department of Biotechnology, School of Life Science and Biotechnology, Adamas University, Kolkata, West Bengal, India; ^2^School of Mechanical Engineering, Vellore Institute of Technology, Vellore, Tamil Nadu, India; ^3^Department of Zoology, Fakir Mohan University, Balasore, Odisha, India; ^4^Institute for Skeletal Aging and Orthopedic Surgery, Hallym University Chuncheon Sacred Heart Hospital, Chuncheon-si, Gangwon-do, Republic of Korea

**Keywords:** ChatGPT, Chatbot, medical use, large language models, AI

## Abstract

The release of ChatGPT has initiated new thinking about AI-based Chatbot and its application and has drawn huge public attention worldwide. Researchers and doctors have started thinking about the promise and application of AI-related large language models in medicine during the past few months. Here, the comprehensive review highlighted the overview of Chatbot and ChatGPT and their current role in medicine. Firstly, the general idea of Chatbots, their evolution, architecture, and medical use are discussed. Secondly, ChatGPT is discussed with special emphasis of its application in medicine, architecture and training methods, medical diagnosis and treatment, research ethical issues, and a comparison of ChatGPT with other NLP models are illustrated. The article also discussed the limitations and prospects of ChatGPT. In the future, these large language models and ChatGPT will have immense promise in healthcare. However, more research is needed in this direction.

## 1. Introduction

The appearance of ChatGPT has initiated new thinking about artificial intelligence (AI)-based Chatbot technology and has drawn public attention throughout the globe. The tool was launched on November 30, 2022 and it was popularized very quickly among the students and researchers after it was launched. After ChatGPT appeared, several questions came into everybody's mind about ChatGPT. The first question was can they write “smart essays”? Stokel-Walker (2022) has written something new in this direction and commented that it provides prompt responses to the user; the AI-enabled Chatbot can create sound and intelligent text, write exam-style questions, and do homework assignments. Another article by Castelvecchi (2022) has informed that ChatGPT can write meaningful lines of programming code like AlphaCode. However, Li et al. (2022) have shown that AlphaCode can generate millions of various programs and can filter and cluster those programs. Similarly, Kashefi and Mukerji (2023) stated that ChatGPT could write the programming code of numerical algorithms. Recently, Perkel (2023) has reported that this AI-based large language model (LLM) can annotate and debug code. It can interpret software from one language to another programming language.

However, it is needed to provide clear definitions of Chatbot and ChatGPT. A Chatbot is an automated computer program that simulates conversations with human users. These Chatbots leverage natural language processing (NLP) and machine learning (ML) algorithms to comprehend and respond to user inquiries conversationally and intuitively. ChatGPT is an AI derived Chatbot that uses Deep learning (DL) technology for information processing. This Chatbot has been introduced and developed by OpenAI Inc (San Francisco, CA, USA), which utilizes NLP to create text-based responses to user input. This Chatbot is constructed on the generative pre-trained transformer (GPT) architecture, a large-scale language model (also called LLM) that can execute various language-based tasks. However, in medicine, the question was: will ChatGPT change medical care? An editorial published in Nature Medicine has answered the question and confirmed that this LLM would help patients' care, quality of life, and better healthcare delivery. It has immense potential to help with clinical needs. They concluded that the evaluation of language models would transform the medical field (Editorial, 2023b). Similarly, Khan et al. (2023b) provided their viewpoint about the role of the AI-enabled Chatbot clinical management and medical education. Graber-Stiehl (2023) argues how this tool can be used as a therapist, like a mobile mental-health app.

Similarly, Yang (2023) discussed how this tool is used in teaching. Therefore, it can be used for medical teaching also. The tool is now prevalent, and scientists are exploring its functions in diverse areas such as teaching and education, computer programming, different fields of health care and cultural heritage. However, ChatGPT has come to the forefront of all recent Chatbot technologies powered by DL-based language models.

In the Chatbot technology, computer program has been developed for the Chat with the human user. It can distinguish questions and provide automatic responses. Modern Chatbot technologies use more specific branches of AI Such as DL and NLP. It can interact with human users through the visual languages, in written and spoken form (Adamopoulou and Moussiades, 2020b; Haque and Rubya, 2023). The Chatbot technologies are described as AI-based example of the Human-Computer Interaction (HCI). Chatbots are also famous as artificial conversation entities, digital assistants, interactive agents, or smart bots.

A literature search was conducted to cover relevant studies on Chatbots and ChatGPT in medicine. PubMed, Scopus and Google Scholar were used, focusing on articles published between September 2021 and July 2023. Primary search terms included variations of “Chatbot,” “ChatGPT,” “medical Chatbot,” “healthcare Chatbot,” “artificial intelligence,” and “machine learning.” The search strategy was adapted to each database's requirements and functionalities. The selection criteria for articles focused on Chatbots and ChatGPT in medicine or healthcare. Articles which discussed the use of Chatbots in patient communication, diagnosis support, and other medical tasks, as well as the benefits, limitations, challenges, and future directions of Chatbot technology were considered. The selected articles were analyzed for utilization, performance, and outcomes of Chatbots and ChatGPT in medical applications. Relevant information was extracted and summarized for an overview of current research in this area. The article revealed valuable information regarding the potential use of Chatbots and ChatGPT in medicine, including their implementation and effectiveness.

This article discussed the overall landscape Chatbot and ChatGPT in two main headings. We discuss the overview of Chatbots and then the role of ChatGPT in medical science. In the first section, to illustrate the general idea of Chatbots, we have presented the overview, evolution, architecture, technology used and the medical use of Chatbot technologies. In the second section, to discuss ChatGPT, we illustrated its application in medicine, architecture and training methods, medical diagnosis and treatment, research ethical issues associated with ChatGPT in medicine, and a comparison of ChatGPT with other NLP models used in medicine. We also discussed limitations and prospect.

## 2. Chatbot: an overview

**Figure 1:**
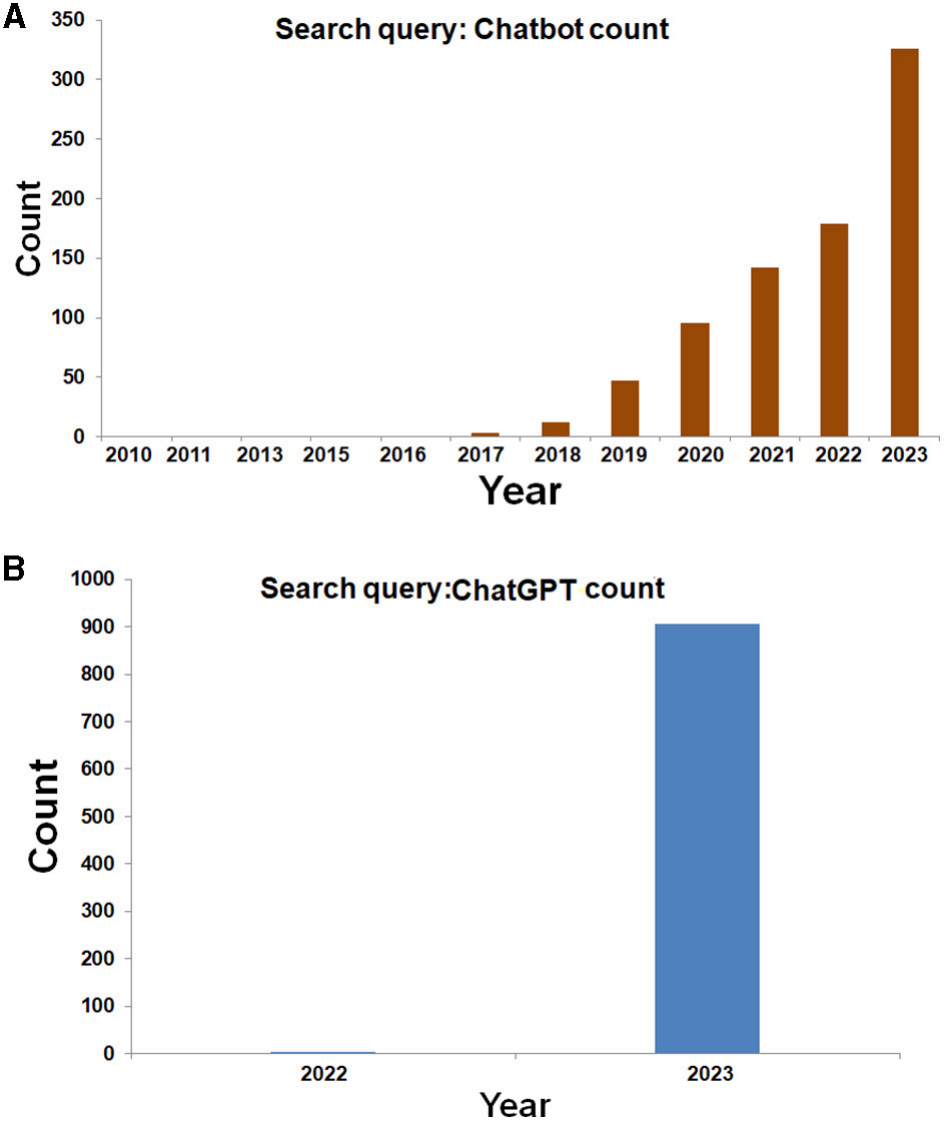
Publication search result of Chatbot and ChatGPT from PubMed. We used the keywords “Chatbot” and “ChatGPT” during the search (title/abstract search). **(A)** Publication search result of Chatbot from PubMed. The search result shows that the number of publications has increased daily since 2018. **(B)** Publication search result of ChatGPT from PubMed. The search result shows that the number of publications of ChatGPT has increased very quickly after its release in November 2022 by OpenAI.

Several scientists provide a simple definition of the Chatbot. They explained that it is a computer program developed to successfully perform conversations between human users and computer programs using the internet. Although it is an artificial conversation entity, it uses AI to respond like an intelligent entity. It can respond through text or voice and understand one or more human languages (Adamopoulou and Moussiades, 2020a). Chatbots became more popular over time, and user numbers were increasing consecutively. In 2015, there were about 3.15 billion human users. In 2016, the number of human users increased and became 3.39 billion. In 2017, the number of users was 3.58 billion (Suta et al., 2020). Similarly, we performed a PubMed search to understand the Chatbot's popularity among scientists for publication, and we found 652 publications with the “Chatbot” and 572 publications with the “ChatGPT” keyword (Figures 1A, B). At the same time, we found an increasing trend of yearly publications for both keywords. Therefore, we can infer that the popularity of Chatbot technology is increasing.

**Figure 2:**
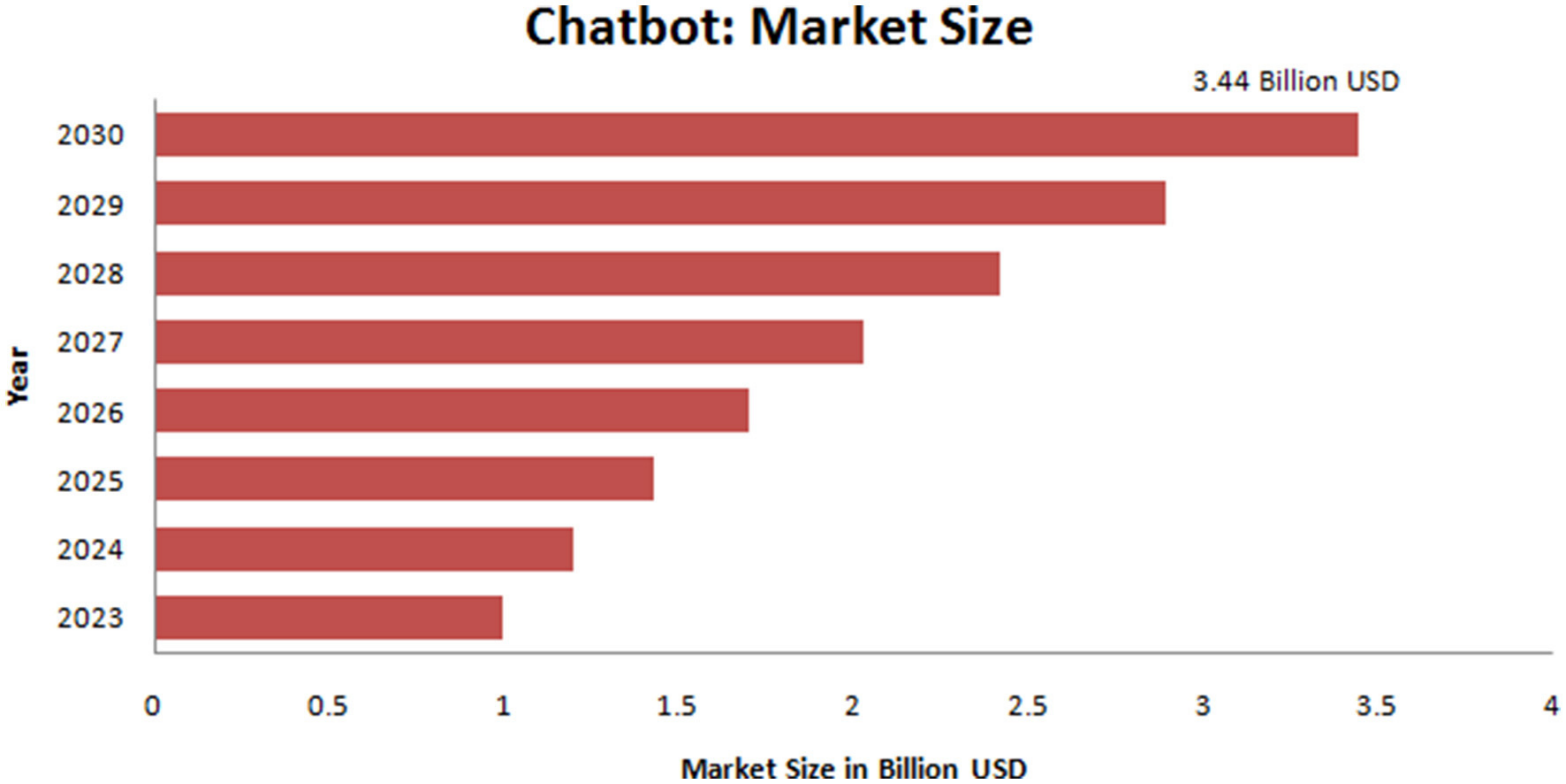
The market size of Chatbot. The calculated market size of Chatbot was about 1 billion in 2013, and the expected market size in 2030 is about 3.44 billion.

Chatbots were developed using pattern matching with the simple “Q & A” format to match and carry out the human-like conversation (Smutny and Schreiberova, 2020; Kuhail et al., 2023). Now Chatbots are assisting in performing different functions such as answering questions, performing a task, discussing a specific topic, or providing information. Today's Chatbot landscape has broader perspective. Chatbots are not associated as a single category but fall along a broader spectrum. Several scientists have provided a classification from time to time regarding their properties such as input, web-based service, messaging channels, etc. (Smutny and Schreiberova, 2020). Some other classifications include voice bot, hybrid Chatbot, social messaging Chatbot, menu-based Chatbot, skills Chatbot, keyword-based Chatbot, transactional bot, No code or low code Chatbot, etc. (Table 1). Recently, Chatbots have shown promise to revolutionize the educational landscape by supporting educators and personalizing learning activities with other activities (Kuhail et al., 2023). Chatbots provide quick and convenient support to users responding specifically to their questions. Therefore, Chatbots have immense promise; presently, several organizations are trying to create industrial solutions. Due to the performance of its different activities and popularization, there is a considerable market value in the Chatbots industry. The Chatbot industry value in the USA was calculated as 113 million USD. It is expected to multiply, and in 2024, it will reach ~1 billion USD (Kuhail et al., 2023; Statista, 2023). However, the Chatbot market size is growing day by day quickly (Figure 2).

**Table 1:** Some Chatbot types and their features.

**Sl. No**.	**Chatbot types**	**Algorithms used**	**Features**
1.	Voice bot	Natural language understanding (NLU)	It applied voice-to-text and text-to-speech communication channel powered by AI and an interactive voice response (IVR) system with their voice
2.	Hybrid Chatbot	Machine learning/natural language processing (NLP)	The accurate composite of Chatbot and live chat that combines the best of both domains
3.	Social messaging Chatbot	Self-learning ML models, and natural language processing (NLP)	The social media edges and interface by deploy of AI algorithm across all of their user
4.	Menu-based Chatbot	Natural language processing (NLP)	It is based on menu-driven navigation, follow a fixed decision tree that is displayed to the end in the form of clickable buttons or menus
5.	Skills Chatbot	Multinomial naive bayes algorithm	It accomplish a specific set of tasks, and extended its capabilities using pre-defined skills software
6.	Keyword-based Chatbot	Natural language processing (NLP)	It performed customizable keywords and NLP to detect action triggers in the conversation to understand how to respond appropriately to the user
7.	Transactional bot	Symbolic AI and natural language processing (NLP)	Its focus is to complete a transaction and streamline the user experience by offering a quick and easy channel for a single purpose
8.	No code or low code Chatbot	AI and machine learning (ML) algorithms	This allows for speedier application delivery and faster value generation since a graphical user interface (GUI) is available to build and configure; it also suitable for information-collecting Chatbots and those that encourage human interaction

### 2.1. Evolution of Chatbots

Alan Turing first proposed the idea of AI and conceived the term in 1950. He proposed the “imitation game” to understand the machine's capacity, which can be distinguished from humans (Turing, 1950; Zador et al., 2023). In 1966 at MIT, The first Chatbot was developed with the name “ELIZA” by Weizenbaum (1966). It was the first program that was competent in attempting the Turing test. The perspective was to act as a psychotherapist (Weizenbaum, 1966). The program applied pattern matching (Brandtzaeg and Følstad, 2017). After a few years, ELIZA was improved, and a new Chatbot was developed in 1972, which was named PARRY (Colby et al., 1971). Again another new Chatbot was created in 1995, named ALICE. It used a pattern-matching algorithm and was the first Chatbot using the AIML (Artificial Intelligence Markup Language) (Marietto et al., 2013).

**Figure 3:**
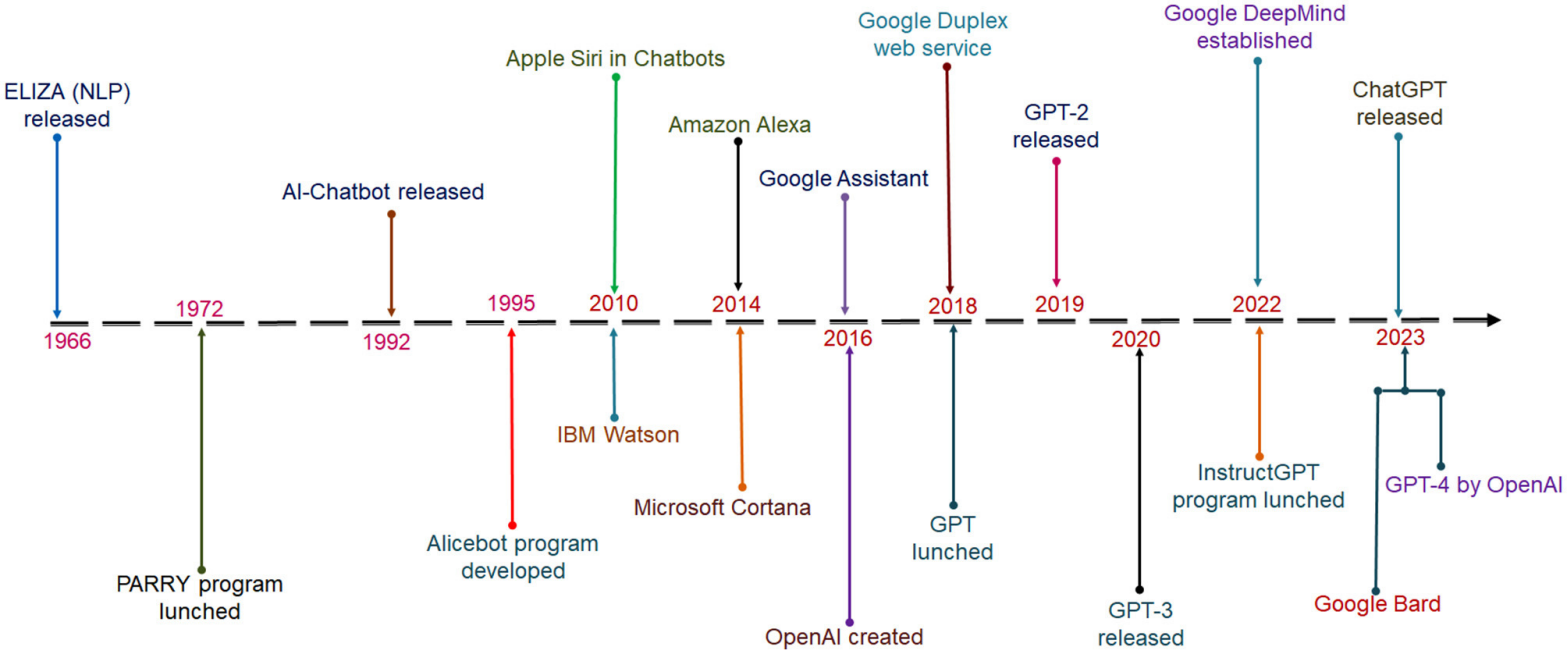
A timeline shows the origin of different Chatbots. The timeline highlighted all the Chatbot from 1966 to 2023.

Several Chatbots were developed from time to time: Apple Siri, IBM Watson, Amazon Alexa, Microsoft Cortana, and Google Assistant. The recently developed famous one was ChatGPT (Figure 3).

### 2.2. Technological and architectural landscape of Chatbots

The essential components or technology used for Chatbots are Pattern Matching, Natural Language Understanding (NLU), Artificial Intelligence Markup Language (AIML), Latent Semantic Analysis (LSA), Natural Language Processing (NLP), Chatscript, entity, RiveScript, contexts, etc.

In pattern matching, the input text was compared with the text components of a database. It used input (stimuli) sentence and output (response). Eliza and ALICE were developed using pattern matching (Dale, 2016). AIML is an XML-based language based on pattern matching or pattern recognition concepts. It uses tag-based basic dialogue called categories (<category>). ALICE has used the AIML. There are various training methods for Chatbots, each with its own strengths and weaknesses. One common approach is the rule-based method, which uses a set of rules to guide the Chatbot's responses. This can be effective for simple use cases but may not handle complex user queries, and scaling can be difficult. Another procedure is the machine learning method, which involves training the Chatbot on a large dataset of conversations using algorithms. This method can handle complex queries and improve over time, but it requires a significant amount of training data and can be computationally expensive. The third technique is the hybrid approach which combines rule-based and ML methods for a more robust Chatbot. The Chatbot follows the rules for simple questions and uses ML for complex ones. This process can handle a wide range of user queries and improve over time; however, it requires expertise in both methods. Real-life illustrations include Google Assistant, which works on a hybrid methodology and provides users with personalized responses. It has the capability to process a vast array of user queries and can enhance its performance over time by assimilating more data. Secondly, we have Amazon Alexa, which employs ML to comprehend user queries and furnish personalized responses. It is proficient in handling intricate queries, and its performance can be optimized by learning from more data over time. Chatbots based on the three training methods mentioned above offer 24/7 availability, cost-effectiveness, and consistency in responding to user queries. However, they may lack empathy, have a limited understanding of complex queries, and are limited by technical constraints.

**Figure 4:**
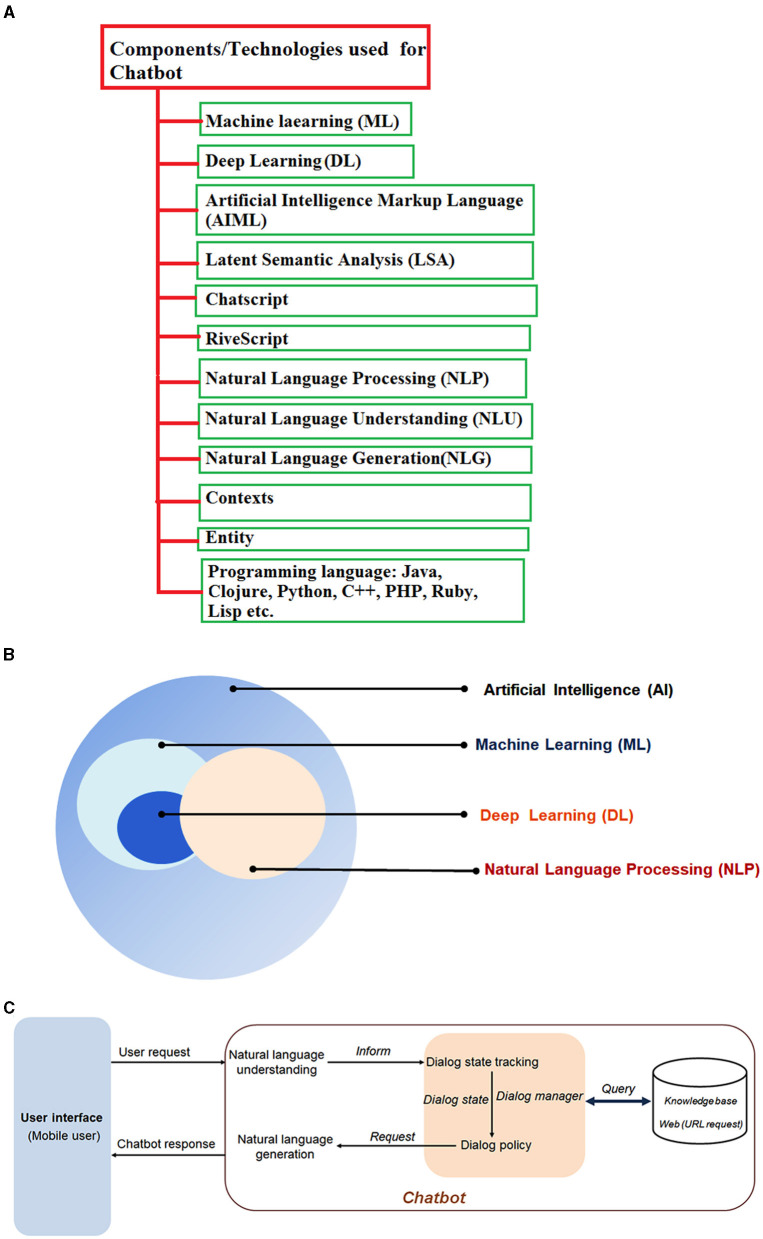
A schematic figure depicts Chatbot's architecture and different components. **(A)** A schematic diagram shows the different components of Chatbot. **(B)** A schematic diagram indicates the relation of AI, ML, DL, and NLP. **(C)** A diagram represents the figure that depicts Chatbot's architecture and process flow.

The essential constituents of a Chatbot system encompass a User Interface (UI), NLP, ML and DL, Dialog Management, and Integration. The UI interacts with the user and it can either be a web interface, a mobile app, or a messaging platform like Facebook Messenger or WhatsApp. NLP component, on the other hand, is responsible for comprehending the user's input and extracting relevant information. It applies techniques such as tokenization, part-of-speech tagging, and named entity recognition to evaluate the user's text. Modern-day Chatbots rely on the use of NLU and Natural Language Generation (NLG) to recognize and respond to users. This is achieved by leveraging the ML and DL elements of AI, which provide responses based on user interactions. The knowledge base of the Chatbot serves as a repository of information which it can utilize in answering user queries. This base encompasses a variety of forms, including databases, sets of rules, or corpora of text. The Dialog Management component assumes responsibility for overseeing the conversation flow and generating appropriate responses based on the user's input and context. It employs a range of techniques, namely state machines, decision trees, or DL models. Finally, the Integration component enables the Chatbot to establish connections with external systems, such as APIs, databases, or other Chatbots. The Chatbot can be developed using cutting-edge programming languages, including but not limited to C++, J, Python, Java, Lisp, PHP, Ruby, Clojure, or any other programming language with similar capabilities (Figures 4A–C).

#### 2.2.1. Real-world example of training method

Let us consider an instance to comprehend the architecture. It is assume that we possess a Chatbot pertaining to the current weather conditions, where the Chatbot would furnish the output utilizing the following process.

User input: “What's the weather like today?”

NLP engine (having NLP component and the ML, DL component): proceeds to analyze the user input and recognizes the user's intent, which happens to be a request for information regarding the current weather conditions.

The backend system (comprising Dialog Management component and the Integration component): is prompted to retrieve the latest weather data corresponding to the user's location.

User output: the Chatbot formulates an informative response, detailing the current atmospheric conditions for the user's location.

#### 2.2.2. Advantages of training method

The Chatbot, especially GPT-3 might build a question dataset from specific prompts using supervised learning, and it can fine-tune it. The model provides feedback by the ranking of responses. It uses Reinforcement Learning. There are several advantages: first, it supports the learners by answering questions. Second, it can process complex instructions. Third, it can provide diversified responses.

#### 2.2.3. Limitations of training method

Several limitations have been observed. Sometimes, it provides incorrect answers. At the same time, the information provided by GPT-3 may be biased. It also provides a substantial plagiarized answer. The answers of the same query may vary from user to user.

### 2.3. Chatbot in medicine

Chatbot technology can be used in the medical industry to improve patient care by giving patients 24 h medical advice and assistance. Chatbots that simulate human-like speech are designed to help people manage their health and communicate with their healthcare providers. The workflow, monitoring, diagnosis, treatment, and promotion of good health are other areas where Chatbots might be helpful. Chatbots have made healthcare services more readily available, practical, and effective (Chow and Sanders, 2023). However, other challenges include the need for human aspects in healthcare and problems with accuracy and dependability (Xu et al., 2021). Chatbots are revolutionizing healthcare by giving patients 24/7 access to medical information, setting appointments, and gathering patient data. Healthcare Chatbots that offer exceptional, individualized care have improved patient care (Safi et al., 2020). The capacity of Chatbots to offer patients immediate support and assistance is one of the reasons why the healthcare sector is seeing an uptick in their use. The NLP, decision trees, and ML techniques can all be used to build and develop medical Chatbots (Kidwai and Nadesh, 2020). From a medical point of view, there are three broad varieties of medical Chatbots: informational, diagnostic, and administrative (Safi et al., 2020). Medical Chatbots are becoming a more popular source of health literacy among users. It can take time to assess the worth and efficiency of medical Chatbots, though, several techniques, such as user testing, surveys, and data analysis, can be used to evaluate the usefulness and usability of medical Chatbots (Mokmin and Ibrahim, 2021).

At the same time, researchers believe the healthcare sector's AI-based mode can lower the growing carbon footprint (Wolf et al., 2022; An et al., 2023; Das and Chandra, 2023). Chatbots can diminish the carbon footprint of healthcare facilities by decreasing the necessity for patients to commute to hospitals for minor concerns and uncertainties. Carbon emissions can be substantially reduced through remote consultations for each appointment. So, chatbots can result in a decline in energy-intensive processes and transportation emissions related to healthcare provision. Furthermore, predictive analytics driven by AI can optimize the allocation of resources, thus minimizing excessive utilization of facilities and resources. Moreover, AI can facilitate the early identification and treatment of diseases, potentially lessening the environmental impact of advanced-stage treatments. Consequently, healthcare models driven by AI not only enhance patient outcomes but also contribute to a more environmentally friendly and sustainable healthcare system.

Several Chatbots are applied in medical science to provide some solutions in the medical field from time to time, such as Smart Chatbot, ChatGPT, EQRbotChatbot, Ask Rosa, TanaChatbot, Vickybot, COVID-Bot, Otis Chatbot, SnehAIChatbot, Anti-TB Chatbot, and ChatbotWakamola (Table 2). GYANT, a healthcare Chatbot that diagnoses patients and administers medication, and over-the-counter (OTC) Chatbots, which recommend nutritious meals and activities, are two prominent examples of healthcare Chatbots. One of the issues with employing medical Chatbots is ethical concerns. Numerous academicians and medical professionals have emphasized that Chatbots are not technically capable of diagnosing medical issues or substituting for professional opinions (Parviainen and Rantala, 2022; Haug and Drazen, 2023). Another issue is that patients generally do not trust medical Chatbots due to low confidence in offering reliable guidance or information. Medical Chatbots may use biased algorithms that can be harmful (Sharma et al., 2022). While Chatbot algorithms can perform simple diagnostic procedures, there are some challenges and limitations. Online consultations, other forms of virtual assistance, and other crucial elements could overlook for a reliable result. Despite these challenges and limitations, medical Chatbots have an opportunity to transform many aspects of healthcare. Healthcare providers utilize medical Chatbots to respond the inquiries, create medical records and provide disease information (Chang et al., 2022).

**Table 2:** Different types of Chatbots and their application in medical science.

**Sl. no**	**Different Chatbots**	**Application in medical sciences**	**References**
1.	Smart Chatbot	Support for interactive management in beta thalassemia patients by performing the follow up on their condition and that provide the required assessment information. Finally, it monitors the patients' statuses.	(Alturaiki et al., 2022)
2.	Chatbot (Emohaa)	The conversational agent that provides cognitive support through CBT-Bot exercises and guided conversations for mental healthcare and support in China.	(Sabour et al., 2023)
3.	ChatGPT	The speciliased decision support tool specifically used as support tool for breast tumor board. Additionally, this tool promising for performance and feasibility for the use in patient management.	(Sorin et al., 2023)
4.	Chatbot (EQRbot)	It used to generate explanations for patients' treatment advice specifically for as a pattern of explanation-question-response interactions between agents in comprises multiple premises that can be interrogated to disclose additional data.	(Castagna et al., 2023)
5.	Ask Rosa	Used to identify best practices for future patients about hereditary breast and ovarian cancer for future patient-focused chatbots. Siglen et al., showed the participatory methodology in combination with an iterative approach ensured that the patient perspective was incorporated at each level of the development process.	(Siglen et al., 2022)
6.	ChatGPT/GPT-4	Applied for support as intensive care unit medicine. More exclusively it meant for range from knowledge augmentation, device management, clinical decision-making support, early warning systems, and establishment of intensive care unit (ICU) database.	(Lu et al., 2023)
7.	Tana (Chatbot)	Used as a complement to care by health personnel during the COVID-19 period especially in times of high demand or constrained resources at the Hospital Italiano de Buenos Aires.	(Rizzato Lede et al., 2022)
8.	Chatbot	Mental health and self-care discovery of patients along with the viability of these kind of crowdsourced methods are in digital healthcare. It also implementing the self-care method discovery directly within the conversation.	(Moilanen et al., 2023)
9.	Chatbot	Implemented as behavioral health during the COVID-19 pandemic; it is an algorithm-based, automated, and interactive artificial intelligence conversational tool that uses natural language understanding to employ users by presenting a series of questions with simple multiple-choice answers.	(Jackson-Triche et al., 2023)
10.	Chatbot	The evidence-based mobile health implanted conversational agent to identify facilitators and barriers to incorporating mental health apps in treatment planning for adolescents with depression and anxiety, in support for cognitive behavioral therapy.	(Nicol et al., 2022)
11.	Vickybot	Applied in anxiety-depressive symptoms and work-related burnout in primary care and health care professionals during the COVID-19 pandemic. It used to screening, monitoring, and reducing anxiety-depressive symptoms and work-related burnout, and detecting suicide risk in patients from PC and health care workers.	(Anmella et al., 2023)
12.	Chatbot (COMPASS)	Improving the well-being of adolescents with type 1 diabetes patients by using the self-compassion Chatbot during the COVID-19 pandemic. It included the problem-solving and integration with diabetes technology to support self-management; creating a safe peer-to-peer sense of community; and broadening the representation of cultures, lived experience stories, and diabetes challenges.	(Boggiss et al., 2023)
13.	ChatGPT	This tool used in prediction of forthcoming diabetes technology by patient's outcomes.	(Huang et al., 2023a)
14.	COVID-Bot	Screening, design and development of COVID-19 vaccination and its confirming status. The Chatbot formulated by design science research (DSR) process, for creating a new scientific artifact to combat the spread of COVID-19 among university students.	(Okonkwo et al., 2022)
15.	Chatbot	The text-based conversational agents applied for mental health care and future assessment. This digital tool has potential strengths and limitations.	(Schick et al., 2022)
16.	Chatbot	The text-based interaction with human users via a conversational interface used in stress and health-related parameters in a stressed sample. The tool comprised flexible time points of the intervention units and the ecological momentary assessments, reminder messages, and the opportunity to postpone single units.	(Schillings et al., 2023)
17.	Chatbot (Otis)	The mixed methods of cognitive behavioral therapy (CBT)-based Chatbot applied for patient's health anxiety management for adults in New Zealand during the COVID-19 pandemic.	(Kim et al., 2021)
18.	SnehAI (Chatbot)	This is used for young people's sexual and reproductive health in India. In board sense it purposefully designed for social and behavioral changes in India, to provide a private, non-judgmental, and safe space to spur conversations about safe sex and family planning, and offers accurate, relatable, and trustworthy information.	(Nazareth et al., 2021)
19.	Chatbot	Used for COVID-19 contact tracing during a surge in San Francisco, California assigned by telephonic interview.	(Asensio-Cuesta et al., 2021)
20.	Chatbot	It affects smile and speech in patients having Parkinson's disease. Precisely this study used to investigate whether smile and speech features could predict motor, cognitive, and mood status of patients.	(Kataoka et al., 2021)
21.	Anti-TB Chatbot	It provides information about the disease and its treatment to people vulnerable to TB in South Korea, and helps to understand the factors that predict TB acceptance by the population by the extended Technology Acceptance Model (TAM).	(Thirunavukarasu et al., 2023b)
22.	Chatbot	It can help to identify patients at high risk for hereditary cancer syndromes before routine care appointments in women's. Subsequently it can effectively provide cancer risk assessment, engage patients with educational information, and facilitate a path toward preventive genetic testing.	(Xu et al., 2021)
23.	Chatbot (Wakamola)	This tool collect data from a population about sociodemographics, diet patterns, physical activity, BMI, and specific diseases to interact with users in mobile health apps used for both individual and social perspectives.	(Pham et al., 2022)
24.	Chatbot	It used to educating patients with lung cancer and their caregivers in Japan. This Chatbot designed to improve the knowledge of symptom management among patients.	(Schmidlen et al., 2022)
25.	Chatbot (Med-PaLM 2)	The most trained Chatbot by Google health research teams with medical knowledge, can answer questions and summarize insights from a variety of dense medical texts. Presently, “expert” level on U.S. Medical Licensing Exam-style questions.	(Chaix et al., 2022)
26.	Chatbot	This Chatbot predominantly applicable in health care, with the capability for complex dialog management and conversational flexibility in in medicine. It helps to improve the quality of care for patients, rebalance the workload for clinicians, and revolutionize the practice of medicine.	(Goldenthal et al., 2019)
27.	Chatbot	It generally study's the impacts in digital psychiatry, AI chatbots enabled to assist with psychiatric diagnoses, symptom tracking, disease course prediction, and psychoeducation.	(Kadariya et al., 2019)
28.	Chatbot	This Chatbot support to increase uptake of cascade genetic testing. It also describes the proband's result, associated disease risks, and recommended management and captures whether the user is a blood relative or caregiver, sex, and relationship to the proband.	(Moutsana Tapolin et al., 2023)
29.	Chatbot	It gathers real-life data of patients suffering from primary headache disorders. By personalized text messages clinicians have collect patient-reported outcome for screening the impact of a disease.	(Sabry Abdel-Messih and Kamel Boulos, 2023)
30.	Chatbot	Evaluated the Chatbot usage and their ability to deliver information to patient post-ureteroscopy at the University of Michigan. Effective for the concerns surrounding common symptoms and quick access to information for non-emergent issues.	(Currie et al., 2023)
31.	Chatbot (kBot)	The personalized Chatbot for asthma self-management; it is android application with a frontend chat interface capable of conversing in both text and voice, and a backend cloud-based server application that handles data collection, processing, and dialogue management.	(Yeo et al., 2023)
32.	Chatbot	The conversational web-based Chatbot used to disseminate COVID-19 advisory information developed by the University of Ulster's Chatbot Usability Questionnaire (CUQ).	(Haman et al., 2023)

## 3. ChatGPT: AI and NLP enabled, newly developed Chatbot

### 3.1. Overview

The enormous language model-based question-and-answer system known as ChatGPT has created business and academic interest (Guo et al., 2023). The OpenAI Company in the USA developed ChatGPT. Presently, OpenAI has released ChatGPT plus which is a subscription plan for ChatGPT. OpenAI is an AI-based research laboratory that developed another famous Chatbot, GPT-4 (Lee et al., 2023; Sanderson, 2023). ChatGPT is a platform that can perform language translation and answer simple questions. On the other hand, GPT-4 is more intelligent and can comprehend images along with the text. As a state-of-the-art AI system, ChatGPT can produce content with a natural conversational flow and react to requests fast. As a result, for example, poetry, children's books, and fan fiction can all be created. ChatGPT has undergone considerable testing, and the results have been positive in various industries, including software testing, education, and healthcare (Roberts et al., 2020). In-depth human evaluations and linguistic analyses of ChatGPT-generated content in comparison to that produced by people have been carried out by researchers to understand the traits of ChatGPT's replies, better the differences and gaps from human experts, and the future directions for huge language models (Anderson et al., 2023; Jalil et al., 2023; Jang and Lukasiewicz, 2023). The capability of ChatGPT to generate text that resembles human-written language may revolutionize medical practitioners' interactions with patients, data analysis, and research. ChatGPT's integration into the healthcare system and its opportunities and challenges in medicine have been highlighted (Hügle, 2023; King, 2023; Loh, 2023).

### 3.2. Natural language processing (NLP) and its application in medicine

NLP, a subfield of AI that evaluates human language, has been used in several medical specialties. In ophthalmology, NLP has extracted specific language from free-text notes for quantitative analysis, such as visual acuity (Chen and Baxter, 2022). In databases of clinical notes, pathology reports for diabetes, schizophrenia, cancer, and cardiovascular illness have been analyzed using ML-based NLP. NLP has been used extensively, whether using DL or a conventional method (Mollaei et al., 2022). In the study and therapy of breast cancer, NLP has been used to interpret pathology reports, discover penetrance records for breast and other cancer susceptibility genes, and mine electronic medical data (Hughes et al., 2020). NLP has been used in traditional Chinese Medicine to extract information from unstructured text data (Zhang et al., 2022). Clinical neurosciences have used NLP easily to synthesize literature, extract data, identify patients, automate clinical reporting, and forecast outcomes. By utilizing the enormous amount of text data now available through big data analytics and predictive AI, NLP offers the potential to advance healthcare (Zhang et al., 2022).

### 3.3. Overview of ChatGPT's architecture and training methods

**Figure 5:**
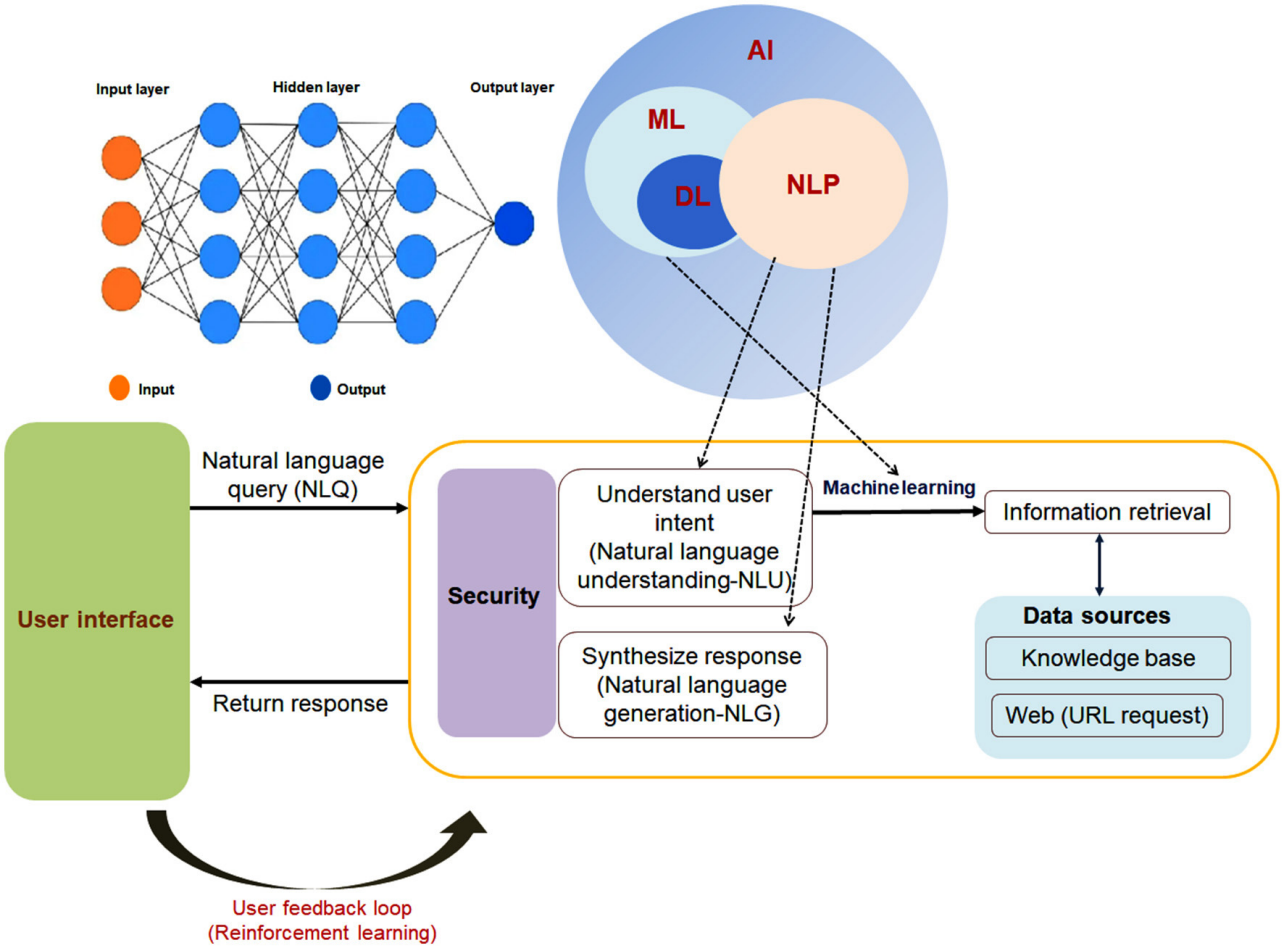
A schematic figure depicts the architecture of AI-based ChatGPT. It also depicts the process flow of ChatGPT. It shows that it uses DL-based language models. The modes have been designed to process and generate responses using NLU and NLG.

The architectural foundation of ChatGPT is a transformer model, a class of neural networks that excels at tasks requiring NLP. The capacity of ChatGPT to use DL techniques in producing responses to text input, that are human-like and far superior to those of any other AI model has attracted a lot of attention (Wen and Wang, 2023). It has a unique architecture in terms of processing based on the neural network that handles sequential data. It uses DL-based language models. The models have been designed to process and generate responses using NLU and NLG for any sequence of characters that make meaning from various spoken languages, mathematical equations, and programming languages (Figure 5). A significant volume of material from books, journals, and websites is used to train the model using unsupervised learning techniques. The user interface is connected with middle-layer technologies. However, the model learns to anticipate the next word in a sentence based on the words before it during training. This method allows the model to learn the significance and organization of language (Tampuu et al., 2020). The capacity of ChatGPT to respond logically and contextually suitable to various text questions is one of its primary features. Sophisticated language modeling methods and a sizable and varied training dataset are utilized to perform this function. By training ChatGPT on a smaller and more targeted dataset, specific tasks like sentiment analysis or question-answering can be made better (Iversen et al., 2014). ChatGPT's progress is significant for the study of NLP in general, as it has the potential to radically change how consumers interact with computers and other digital devices. Language models may grow even more sophisticated in the future, given how constantly evolving language model design and training methods are (Cherubini and Dinh, 2023; Shoaib et al., 2023).

### 3.4. Use of ChatGPT in medical diagnosis and treatment

Medical diagnosis and treatment are among the most significant application areas for ChatGPT. But whether it is trustworthy and truthful in these fields is still debatable. Numerous researches have examined the effectiveness of ChatGPT in treating patients and identifying illnesses (Huh, 2023). After studying ten published case reports from Korea, ChatGPT successfully identified three cases when given the patient's symptoms, findings, and medical history in a single examination (Hariri, 2023). After adding laboratory, pathological, and radiographic data, the accuracy rate rose to seven out of ten. ChatGPT was employed in a different study to provide information on cirrhosis and hepatocellular cancer (Yeo et al., 2023). Even though the questions and answers contained a lot of information regarding both illnesses, it was discovered that only a few were regarded as complete. ChatGPT still has issues, such as a tendency for biased responses and the ability to perpetuate undesirable linguistic patterns, despite having the potential to aid medical decision-making and instruction. Therefore, more research is required to assess its validity and precision in medical diagnosis and therapy (Huang et al., 2023b).

A recent experiment published in JAMA tested ChatGPT based on GPT-4 for diagnostic accuracy in complex medical cases. The findings indicate that ChatGPT can accurately identify correct diagnosis in almost 40% of cases, as its primary suggestion, and also deliver the correct diagnosis response as one of its potential advances in two-thirds of the remaining cases tested (Meck, 2023). In another study, Liu et al. (2023) demonstrated the efficacy of ChatGPT in producing clinical letters, radiology reports, medical notes, and documentation for clinical purposes. They observed that it could also furnish automated scoring, instructional assistance, enhanced individualized learning, research aid, generation of clinical scenarios, swift information retrieval, translation, clinical decision support, and personalized medicine. Further, ChatGPT provided a higher percentage of factual and accurate responses, thereby rendering it a more secure and precise instrument for various tasks. Similarly, Ali et al. (2023) have informed researchers in the digital health field that ChatGPT can prepare a clinical letter for the patient. Although ChatGPT provides vast information about medical diagnosis and treatment, several scientists have raised the question about the misinformation provided by ChatGPT (De Angelis et al., 2023). However, more accurate, next-generation, error-free ChatGPT is needed in medical diagnosis and treatment. The domain-specific next-generation, error-free ChatGPT might solve the problem in the future (Chatterjee et al., 2023; Pal et al., 2023).

### 3.5. Use of ChatGPT in medicinal research and other purposes

Along with medical diagnosis and treatment, medical service providers are trying to use ChatGPT to aid medical laboratories, find potential research topics, and inform medical students, doctors, nurses, and other healthcare professionals about updates and new developments in their fields (Dave et al., 2023; Jeblick et al., 2023; Sedaghat, 2023). It has been used in different areas of medical science by researchers. The applied areas are clinical toxicology, medical imaging, medical education, clinical management, cirrhosis and hepatocellular carcinoma, medical report generation, diabetes research, etc. (Table 3). ChatGPT has been used in other fields, such as essay writing, programming, code debugging, teaching, etc. (Table 4). Now, ChatGPT provides medical domain-specific information. Cocci et al. (2023) informed that ChatGPT provides outcomes for urology patients. They performed a case study using 100 patients' data. They found ChatGPT provides interactive information with 52% correct answers. ChatGPT-derived answers were more pertinent for non-oncology areas (58.5%) than oncology (52.6%). Similarly, Singh et al. discussed how it impacts the ophthalmological area. They illustrated that ChatGPT can write ophthalmic discharge summaries with meaningful generic text. The discharge summary can include the medications, next consultation time, follow-up instructions, etc. (Singh et al., 2023). Simultaneously, Dossantos et al. (2023) wrote an editorial discussing ChatGPT's limitations and applications in ophthalmology. In the same direction, medical researchers discussed ChatGPT's impact on orthopedics, radiology, etc. (Fayed et al., 2023; Jiang et al., 2023).

**Table 3:** ChatGPT and its uses in medical science.

**Sl. no**	**Area of use**	**Remark**	**References**
1.	Clinical toxicology	Used in typical clinical toxicology case of acute organophosphate poisoning.	(Nakaya et al., 2023)
2.	Medical imaging	Different case of error correction in medical radiation science of higher education.	(Ferres et al., 2023)
3.	Medical education and clinical management.	Use for documentation and communication with patients data, decision support, personalized learning and research assistance.	(Khan et al., 2023b)
4.	Cirrhosis and hepatocellular carcinoma	It have an important role as an aide informational tool for physicians and patients to improve outcomes.	(Grünebaum et al., 2023)
5.	Diabetes	It predicts the future of diabetes technology.	(Huang et al., 2023a)
6.	Mental health care	Interpreting human behavior and its possible application for mental health care.	(Reis, 2023)
7.	Cardiology	In cardiology, study perform categorization virtual reality (VR) study using confusion matrix.	(Fayed et al., 2023)
8.	Medicine and radiology	It can potentially affect radiology, including the generation of reports, improved communication with referrers and patients, decision support, and analysis of research data.	(Xue et al., 2023)
9.	Obstetrics and gynecology	Concluded by a series of questions to patients about obstetrics and gynecology through ChatGPT as prompts, it evaluated the model's ability to handle clinical-related queries.	(Darkhabani et al., 2023)
10.	Urological science	It can generate text that explains urological conditions and treatments in a clear and understandable way.	(Uprety et al., 2023)
11.	Orthopedics and sports medicine	In this phenomenon ChatGPT assisted with the tracking activities, evaluating diagnostic images, predicting injury risk, and several other uses.	(Fayed et al., 2023)
12.	Clinical and translational medicine	It directly helps in clinical decision support, clinical trial recruitment, clinical data management, research support, patient education and other fields.	(Xue et al., 2023)
13.	Autoimmunity	By the electronic health records and human like answers it applied as accurate and evidence-based clinical decision support resources.	(Darkhabani et al., 2023)
14.	Cancer care	Used for the extract key content from medical records of patients with cancer, interpret the next-generation sequencing reports, and offer a list of potential clinical trial options.	(Uprety et al., 2023)

**Table 4:** The use of ChatGPT in other areas.

**Sl. no**	**Area of use**	**Remark**	**References**
1.	Essays writing	Create smart text, write exam-style questions, and do homework assignments.	(Stokel-Walker, 2022)
2.	Programming code	Ability to write the code of numerical algorithms.	(Kashefi and Mukerji, 2023)
3.	Programming	Annotate and debug code.	(Perkel, 2023)
4.	Teaching	Tool is used in teaching.	(Adamopoulou and Moussiades, 2020b)
5.	Microbial genomics research	May use microbial genomics research.	(Page et al., 2023)
6.	5G Cellular Network Role	5G cellular network in medical application.	(Janamala et al., 2023)
7.	Regulatory activity	Examination pharmacist licensing in Taiwan.	(Wang et al., 2023c)
8.	Water domain	Application in the water domain.	(Ray, 2023)

### 3.6. ChatGPT or other Chatbot technologies in drug discoveries and development

ChatGPT or other Chatbot technologies help pharmaceutical companies provide information on drug discovery and development, and also other therapeutic areas. One recent article by Savage provides information on how ChatGPT is helping scientists to produce probable drug targets and, thereby, offering helpful information about drug targets. This AI-driven platform is a new way to target discovery by augmenting the integration and relationships provided through the knowledge graphs (Savage, 2023).

At the same time, it has been noted that ChatGPT-derived information helps to identify possible hit compounds for drug discovery. This Chatbot can help identify disease-specific genes, agents, and other potential information. It can predict the pharmacodynamics (PD), pharmacokinetic (PK), and toxicity character of a specific compound, which is extremely helpful for drug discovery and development (Zhao and Wu, 2023). Recently, Heck described that ChatGPT could provide cellular information like HSP70, which might be extremely helpful for drug discovery and development (Heck, 2023). At the same time, researchers are trying to develop the domain-specific Chatbot for drug discovery and development. One example is DrugChat, which can produce molecular graphs of drugs (Liang et al., 2023). It is envisaged that ChatGPT or other Chatbot technologies will help to revolutionize drug discovery and development shortly.

### 3.7. Potential ethical issues associated with ChatGPT in medicine

Clinical practices, medical education, and research could all be transformed by using ChatGPT. When utilized in medicine, it brings up possible ethical issues that must be resolved. ChatGPT needs extensive data to enhance its language generation abilities, but this information often carries sensitive personal details, such as medical records, that require safeguarding to ensure privacy (Ovalle et al., 2023). There are extra safety and security issues to consider because the model might not be able to handle sensitive medical information properly. More reliance on technology is needed since practitioners may stop using their clinical discretion instead of the model's suggestions (Pennestrì and Banfi, 2022; Corsello and Santangelo, 2023). Ethical concerns include the need for informed consent, the likelihood that the model may replace real doctors leading to job losses, and a lack of human interaction in healthcare. However, in a practical scenario, AI can never replace real doctors, lacking human interpretation, emotion, and a sense of accountability. Another ethical concern involves potential biases that may occur in healthcare and medicine. ChatGPT's output can be compromised if the training data is biased toward a particular demographic group. Also, using ChatGPT and other AI tools in medical writing raises ethical and legal issues, namely, a potential violation of copyright laws, complications in medico-legal matters, and the requirement for transparency in AI-created content (Liu et al., 2023). Therefore, it is very clear that the replaceability of human physicians is limited, as they bear huge responsibilities. AI cannot be a substitute for a human doctor. Understanding the potential hazards, restrictions, and ramifications of these technologies' use in the medical sector is crucial for addressing these challenges. Some recommendations and good practices can mitigate the current ethical concerns. These include ensuring appropriate measures to protect patient privacy when utilizing ChatGPT and one such measure is utilizing text ambiguation frameworks to preserve user privacy. The next one is ensuring that the training data utilized to train ChatGPT is diverse and representative of the population to avoid biases in its language generation. Ensuring proper legal measures to safeguard patient privacy while using ChatGPT in healthcare and medicine is also essential. ChatGPT should follow the norms set up by governments worldwide, like HIPAA and GDPR.

Furthermore, access to these technologies could be limited in various ways, like by subscription fees, etc., to create novel forms of inequality and discrimination. When using ChatGPT professionally for academic writing, it is important to exercise caution to avoid socially sensitive content, misinformation, or plagiarism. A detailed explanation of the AI methodology should be provided to highlight the contribution of AI in medical writing or research. This includes AI's ML and DL algorithms, specific model architecture, and any alterations or adjustments made to the AI system. The method should be trained and validated as an AI model. Information on how the model's performance should be measured, such as cross-validation or separate test sets, should also be provided. Data utilized to train and test the AI system must be described, specifying the dataset's source, size, and characteristics and any preprocessing steps taken, such as data cleaning, normalization, or feature engineering. Additionally, ensuring transparency in AI-generated content and avoiding any violation of patents is crucial. At the same time, science should go on, and AI-based Chatbot technologies should be used appropriately for the sake of human society. Therefore, these technologies should be used safely, productively, and outcome-based. The publishers, researchers, and countries' regulatory authorities need to sit together and lay down rules about the use of LLMs ethically. One editorial in Nature recently illustrated the role of scientific publishers in deploying AI in medical writing (Editorial, 2023a). The development of AI language models has substantially advanced AI and has the potential to disrupt current clinical practices in all surgical and clinical specialties of medicine. These technologies' ethical and social implications must also be considered to ensure their responsible and ethical use (Aggarwal et al., 2021; Saedi et al., 2021; Lennon et al., 2022; Pennestrì and Banfi, 2022; Editorial, 2023a).

### 3.8. Comparison of ChatGPT with other NLP models used in medicine and their evaluation metrics

In one study, NLP was used to predict whether candidates for residency interviews would receive an invitation (Mahtani et al., 2023). The NLP model's average AUROC score of 0.80 indicates its predictability. Another study used a hybrid modeling strategy to abstract CT imaging indications by combining structured data from electronic health records with NLP from radiology reports. The study found that for CT imaging indications, the NLP model could accurately distinguish between surveillance and other causes (Khan et al., 2023a). On the other hand, ChatGPT has been utilized in medical research to produce text-based answers to inquiries. Despite being able to deliver responses that resemble those of a human, it has issues with accuracy and bias. For instance, a recent study discovered that ChatGPT produced biased replies to questions about mental health.

Additionally, ChatGPT needs domain-specific knowledge, so it could not be as accurate as other NLP models in analyzing medical data (Dahmen et al., 2023). Appropriate evaluation metrics and assessment techniques must be devised to measure ChatGPT's efficacy (Vat et al., 2021). In a study, ChatGPT's performance was evaluated in the medical physiology examination of phase I MBBS (Subramani et al., 2023). The accuracy and applicability of the information provided in ChatGPT's responses were assessed using theoretical questions from the study. The findings demonstrated that ChatGPT performed well in giving accurate and pertinent information. ACGME milestones have been employed as evaluation measures in emergency medicine (Salzman et al., 2018). Another study examined three specific evaluation measures to assess clinicians' competence using high-fidelity simulation as an assessment tool (Leuck et al., 2013). Evaluation metrics are crucial for determining whether AI and ML are valuable tools. ML and AI are becoming increasingly popular in radiology and medicine, and the number of studies in this area is overgrowing (Handelman et al., 2019).

#### 3.8.1. Comparison between ChatGPT and other NLP models concerning specific performance metrics

In the research conducted by Wang et al. (2023a), an investigation of LLMs, including ChatGPT, was carried out to evaluate document-level machine translation. Their study indicates that ChatGPT can deliver superior performance in terms of human evaluation when compared to other commercial LLMs or other NLP models that use machine translation systems. The ChatGPT demonstrates a strong aptitude for explicating discourse knowledge. In a different study, Lai et al. (2023) explored the role of ChatGPT as a multidimensional evaluator for text style transfer. They emphasized that ChatGPT achieved competitive correlations with human judgments compared to automatic metrics. Therefore, ChatGPT has competitive correlations compared to other commercial LLMs or other NLP models.

However, there are some examples of the underperformance of ChatGPT. Khondaker et al. (2023) evaluated ChatGPT on various Arabic NLP tasks. This group concluded that ChatGPT consistently underperforms when trained in context (few-shot) compared to much smaller dedicated models fine-tuned in Arabic. Hence, the researchers suggested ample scope for improving ChatGPT, including LLMs.

### 3.9. Statistical analysis of ChatGPT's role in medical science

One of the case studies illustrating the potential of ChatGPT in the healthcare domain pertains to the benchmarking of ChatGPT-4 on the ACR Radiation Oncology In-Training (TXIT) Exam and Red Journal Gray Zone Cases. This particular study aimed to assess the performance of ChatGPT-4 in the specialized field of radiation oncology by employing the 38th ACR TXIT exam and the 2022 Red Journal Gray Zone cases. ChatGPT-4 attained scores of 63.65% and 74.57% on the TXIT exam, thereby underscoring the advantages offered by the latest iteration of the ChatGPT-4 model (Huang et al., 2023a). Furthermore, this study effectively identified the areas in which ChatGPT-4 excelled and displayed weaknesses within the realm of radiation oncology, thus showcasing its potential in medical education for the general public and in facilitating decision-making in the field of radiation oncology. Another noteworthy case study by Ma et al. (2023) pertains to evaluating ChatGPT's capabilities in medical report generation. This particular study proposed the employment of ImpressionGPT, an iterative optimizing framework for radiology report summarization in conjunction with ChatGPT. Remarkably, the proposed model achieved state-of-the-art performance on both the MIMIC-CXR and OpenI datasets without requiring supplementary training data.

Consequently, this study effectively demonstrated the potential of ChatGPT in the context of radiology report summarization. This development could enhance radiologists' efficiency while facilitating improved communication between radiologists and other medical practitioners. Gilson et al. (2023) investigation appraised the efficacy of ChatGPT in addressing questions encompassed within the United States Medical Licensing Examination (USMLE) Step 1 and Step 2 examinations. ChatGPT attained accuracies of 44% and 42% on AMBOSS-Step1 and AMBOSS-Step2, respectively, and 64.4% and 57.8% on NBME-Free-Step1 and NBME-Free-Step2, respectively. This study showcased the capability of ChatGPT in medical education and the evaluation of knowledge. Conducting a trial of a substantial language model (ChatGPT) in general practice alongside the Applied Knowledge Test (AKT) serves as another illustrative example that highlights the possibilities and constraints of AI chatbots in primary care (Thirunavukarasu et al., 2023a). This research scrutinized the merits and demerits of ChatGPT in primary care through the employment of the membership of the Royal College of General Practitioners Applied Knowledge Test (AKT) as a medium. The findings of this study revealed that the average overall performance of ChatGPT amounted to 60.17%, which falls below the average passing threshold of the past 2 years (70.42%). This investigation furnishes evidence that ChatGPT harbors promise in primary care settings, but further research is requisite to enhance its performance. In a separate work, Wang et al. (2023b) performed a case study on the utility of ChatGPT in pharmaceutical development for the creation of a drug to combat addiction to cocaine. In this study, GPT-4 was utilized as a virtual mentor, providing valuable strategic and methodological guidance to researchers focused on developing generative models for potential drug candidates. The main objective of this study was to generate drug-like molecules that possess the desired properties, and by harnessing the capabilities of ChatGPT, this study introduced a fresh and innovative approach to the drug discovery process. So, Chatbots assume the role of facilitators, directing researchers toward inventive methodologies and fruitful avenues for creating efficacious drug candidates.

### 3.10. Limitations and future prospect

ChatGPT has passed the theoretical component of the US medical licensing examination despite needing additional training or in-depth medical education (Au Yeung et al., 2023). But employing ChatGPT and other AI technologies in medical writing raises moral and legal concerns, like the need for transparency in AI-generated work and potential copyright breaches. Even though ChatGPT has a lot of promise to improve patient-centered care in radiology and other medical disciplines, more investigation is needed to determine whether it has any disadvantages. Inaccurate statements and possibly dangerous outcomes have all been reported. Therefore, it is essential to consider the limitations of ChatGPT and other foundation models, mainly when used in the medical sector (Wen and Wang, 2023). The usage of ChatGPT as an embodied conversational agent in a social VR platform illustrates the potential of integrating external frameworks into social VR to create new sorts of collaborative experiences (Numan et al., 2023). It demonstrates how adaptable ChatGPT is as a tool for improving user experiences across various scenarios. In the future, ChatGPT will be highly beneficial in mental health. Participants in a study conducted by the mental health software start-up Koko had access to a therapeutic conversational experience where they could talk to ChatGPT about their mental health issues (Grodniewicz and Hohol, 2023). Chatbots can also offer services and information in public health, such as helping smokers quit (Ogilvie et al., 2022). ChatGPT can deliver individualized and accurate medical information, leading to fewer errors and complications, improved patient outcomes, greater patient involvement, and more affordable healthcare delivery (Li et al., 2023). However, to assess the efficacy of ChatGPT in medicine, more research is required because the examined reports need to be of better quality. Despite this, the usage of ChatGPT in the medical industry has a lot of potential. Future research should thoroughly document its functionality, distribution strategies, and theoretical underpinnings (Payton et al., 2017).

## 4. Conclusions

In conclusion, the application of ChatGPT and other Chatbots in the medical industry has shown promise for improving healthcare and medical education. Medical practitioners have found ChatGPT to be beneficial for various purposes, including research, diagnosis, and patient monitoring. Additionally, it can be an effective tool for raising health literacy, particularly among adolescents and young people. There has been a significant rise in demand for Chatbot-based applications that use AI to check symptoms. These apps help users self-triage based on AI and propose potential diagnoses using human-like interactions. It is also essential to consider ethical concerns, such as the reliability and accuracy of the information provided, the likelihood of discrimination, the need for accountability and openness, and other factors. Chatbots like ChatGPT can improve the effectiveness of healthcare delivery, but they can only partially replace doctors. ChatGPT and Chatbots in medicine are prospective study areas requiring more research and development. Future research should be directed toward progressing and refining increasingly intricate and sophisticated Chatbots that can adeptly handle multifarious medical inquiries and concerns. These bots must furnish personalized recommendations based on the patient's unique data and medical history. It is strongly advised that extensive research should be conducted to thoroughly investigate and scrutinize the potential of Chatbots in amplifying patient engagement and improving patient's quality of life. Further, establishing a collaborative relationship among healthcare providers, technology companies, and regulatory bodies might help to develop future and next-generation Chatbots with accountable features. Here, governmental agencies, computer developers, ethicists, scientists, healthcare professionals, patients, support providers, and different regulatory bodies with their regulators and advocates should come together to determine the most suitable way ahead. However, it may be a very challenging task. The effort will help develop DL-based, next-generation Chatbots that will be error-free, responsible, and more ethical with human expertise to improve patients' quality of life.

## Author contributions

The design, investigation, interpretation, and writing—review of this study was done by CC. Analysis and writing—review was performed by SP. Formal analysis and figure-table prepared by MB. Formal analysis and validation by SD. Validation and fund acquisition by S-SL. All authors have read and approved the manuscript.
